# When Normal Flora Turns Pathogenic: A Pediatric Case of Infectious Eczematoid Dermatitis

**DOI:** 10.7759/cureus.84359

**Published:** 2025-05-18

**Authors:** Itzamar Pastrana Echevarria, Alejandra M Figueroa Moreda, Maria G Rodríguez Martínez, Nestor Sánchez Colón

**Affiliations:** 1 Internal Medicine, Universidad Central del Caribe, Bayamón, PRI; 2 Family Medicine, School of Medicine Medical Sciences Campus, San Juan, PRI; 3 Dermatology, Ponce Health Sciences University, Ponce, PRI

**Keywords:** ear dermatoses, eczematous rash, infectious eczematoid dermatitis, mometasone furoate, opportunistic skin infections, pediatric dermatology, staphylococcus epidermidis

## Abstract

Infectious eczematoid dermatitis (IED) is a rare and often underrecognized inflammatory skin condition, particularly in pediatric patients. We present the case of a previously healthy 12-year-old Hispanic child with erythematous, scaly, and pruritic plaques involving the tragus, antitragus, external auditory meatus and lobule of both ears. The lesions were initially treated with topical fluocinolone acetonide prescribed by an otolaryngologist, without clinical improvement. Upon evaluation in our dermatology clinic, a skin culture revealed *Staphylococcus epidermidis* as the causative organism. The patient was treated with ciprofloxacin, selected based on antibiotic sensitivity, along with mometasone furoate 0.1% cream to address both infectious and inflammatory components. On follow-up, the lesions showed improvement; however, the patient was later lost to follow-up. This case highlights the need to consider IED in children with refractory eczematous eruptions.

## Introduction

Infectious eczematoid dermatitis (IED) is a rare, acute inflammatory skin condition that remains poorly described in the medical literature, with particularly limited documentation in the pediatric population. Although its exact incidence is unknown, likely due to underrecognition and misdiagnosis, IED may be more common in individuals with chronic infections, primary immunodeficiencies, or limited access to healthcare services [1-3].

Clinically, IED presents as an eczematous rash triggered by purulent drainage from an underlying infection. Over time, the affected areas become erythematous, crusted, and scaly [4]. Due to its variable appearance, IED is often mistaken for other dermatologic conditions such as impetigo, allergic contact dermatitis, or tinea infections. This overlap highlights the need for careful clinical assessment, including a thorough patient history and microbiologic testing when appropriate.

The condition is most commonly associated with bacterial infections, particularly Staphylococcus aureus and Streptococcus species [4]. Early identification is crucial, as untreated cases can lead to serious complications such as cellulitis, abscess formation, and even necrotizing fasciitis, a rapidly progressive and life-threatening infection requiring emergent intervention [5].

Management typically involves the application of topical antibiotics, such as mupirocin or fusidic acid, which help reduce the bacterial load and resolve localized infection. In more severe presentations or when deeper tissue involvement is suspected, systemic antibiotics may be necessary to prevent further spread [6].

Given its potential severity and the diagnostic challenges it poses, increased awareness and reporting of IED, especially in pediatric populations, are essential for improving recognition, treatment, and outcomes.

## Case presentation

**Figure 1:**
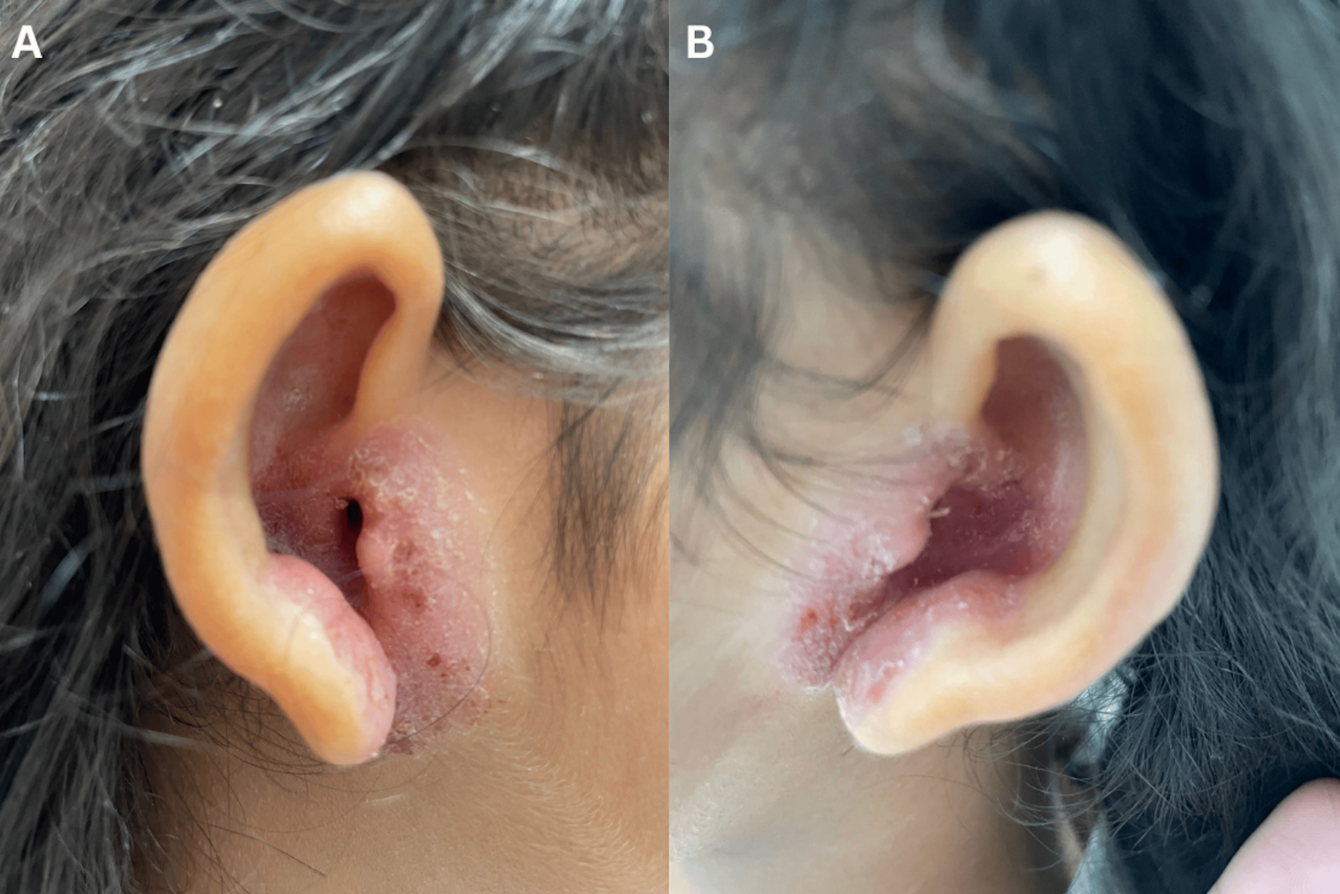
(A) Right ear showing an erythematous, scaly, and mildly thickened lesion involving the tragus, antitragus, external auditory meatus, and lobule. (B) Left ear with similar findings.

We present the case of a previously healthy 12-year-old Hispanic patient who presented with erythematous, scaly, and mildly thickened lesions involving the tragus, antitragus, external auditory meatus and lobule of both ears (Figure 1). The patient reported persistent pruritus in the affected areas. On physical examination, the lesions measured approximately 1.5 to 2 cm in diameter, based on anatomic landmarks (Figure 1). It was characterized by fine scaling, mild lichenification, and superficial excoriations. The patient had previously been treated by an otolaryngologist with fluocinolone acetonide topical solution for 10 days without significant improvement. In view of the persistence of lesions and symptoms, the patient was referred to a dermatologist where an initial skin culture was taken from the right external auditory meatus. The report concluded that the sample was positive for Staphylococcus epidermidis. Dermatologist confirmed IED. Skin culture antibiotic sensitivity revealed susceptibility to ciprofloxacin, for which it was selected as treatment with the addition of mometasone furoate 0.1% cream to cover both infectious and inflammatory components (Table 1). At follow-up, the patient showed improvement with reduced erythema and pruritus but was unfortunately lost to subsequent dermatology follow-up.

**Table 1:** Skin Culture Antibiotic Sensitivity Profile of Staphylococcus epidermidis Isolated From the Right External Auditory Meatus MIC: Minimum Inhibitory Concentration

Antibiotic	MIC (µg/mL)	Interpretation
Azithromycin	>4	Resistant
Ciprofloxacin	<=1	Susceptible
Clindamycin	<=0.25	Susceptible
Daptomycin	<=1	Susceptible
Erythromycin	>4	Resistant
Gentamicin	<=4	Susceptible
Levofloxacin	<=1	Susceptible
Linezolid	<=1	Susceptible
Moxifloxacin	<=0.25	Susceptible
Oxacillin	<=0.25	Susceptible
Rifampin	<=1	Susceptible
Tetracycline	<=4	Susceptible
Trimethoprim/Sulfamethoxazole	>2/38	Resistant

## Discussion

This case highlights a rare presentation of IED in a previously healthy 12-year-old Hispanic patient, with erythematous, scaly plaques localized to the tragus, antitragus, external auditory meatus and lobule of both ears. The lesions were initially treated with topical fluocinolone acetonide without clinical improvement. A subsequent skin culture revealed Staphylococcus epidermidis as the causative organism, supporting the diagnosis of IED.

IED is an uncommon inflammatory dermatosis that arises secondary to purulent drainage or microbial contamination from an adjacent infection site. It is characterized by an acute eczematous reaction, typically featuring erythema, scaling, vesiculation, and superficial crusting [3]. While most documented cases are attributed to Staphylococcus aureus or Streptococcus species, this case is unusual in that it implicates Staphylococcus epidermidis, a coagulase-negative staphylococcus generally regarded as part of the normal skin flora. However, S. epidermidis can act as an opportunistic pathogen, particularly in compromised skin barriers, such as those affected by scratching, excoriation, or inflammation [7]. 

Management of IED requires addressing both the inflammatory and infectious components of the condition [5]. In this case, treatment was guided by the results of the skin culture, which identified Staphylococcus epidermidis as the causative organism. Ciprofloxacin was chosen based on its susceptibility profile, demonstrating a minimum inhibitory concentration (MIC) of ≤1 µg/mL, as well as its accessibility and affordability in the patient’s setting. To manage the inflammatory aspect, mometasone furoate 0.1% cream was prescribed. The patient was instructed to return for follow-up in three weeks; however, they were ultimately lost to follow-up. 

This case emphasizes the clinical relevance of IED in children, a population where the condition is rarely reported. It serves as a reminder that even commensal organisms such as S. epidermidis can contribute to clinically significant disease when skin integrity is compromised. Prompt recognition and appropriate management are crucial to prevent potential complications, including cellulitis or abscess formation. Increased awareness and documentation of such cases can help improve diagnostic accuracy and guide future treatment strategies.

## Conclusions

Clinicians should maintain a high index of suspicion for IED in pediatric patients presenting with refractory eczematous lesions, particularly when localized near anatomical areas prone to moisture and irritation, such as the external auditory meatus and lobule of both ears. A comprehensive approach, including timely microbiologic evaluation and assessment of prior treatments, is essential to differentiate IED from other common dermatoses and to identify potential infectious triggers, even when caused by typically non-pathogenic organisms like Staphylococcus epidermidis. As demonstrated in this case, treatment guided by culture results, in conjunction with anti-inflammatory therapy, can lead to more targeted and effective management. Future studies should explore the full clinical spectrum of IED in children, as well as optimal treatment protocols, to enhance early recognition and prevent progression to severe skin infections.

## References

[1] Pichard DC, Freeman AF, Cowen EW (2015). Primary immunodeficiency update: Part I. Syndromes associated with eczematous dermatitis. J Am Acad Dermatol.

[2] Hoskins S, Skoda-Smith S, Torgerson TR, Boos MD (2020). Eczematous dermatitis in primary immunodeficiencies: a review of cutaneous clues to diagnosis. Clin Immunol.

[3] Trope BM, Lenzi ME (2009). Infective dermatitis. Clin Dermatol.

[4] Yamany T, Schwartz RA (2015). Infectious eczematoid dermatitis: a comprehensive review. J Eur Acad Dermatol Venereol.

[5] Miller JM, Binnicker MJ, Campbell S (2024). Guide to utilization of the microbiology laboratory for diagnosis of infectious diseases: 2024 update by the Infectious Diseases Society of America (IDSA) and the American Society for Microbiology (ASM). Clin Infect Dis.

[6] George SM, Karanovic S, Harrison DA (2019). Interventions to reduce Staphylococcus aureus in the management of eczema. Cochrane Database Syst Rev.

[7] Severn MM, Horswill AR (2023). Staphylococcus epidermidis and its dual lifestyle in skin health and infection. Nat Rev Microbiol.

